# Differences in Sense of Belonging, Pride, and Mental Health in the Daegu Metropolitan Region due to COVID-19: Comparison between the Presence and Absence of National Disaster Relief Fund

**DOI:** 10.3390/ijerph17134910

**Published:** 2020-07-07

**Authors:** Young-Jae Kim, Jeong-Hyung Cho, E-Sack Kim

**Affiliations:** Department of Physical Education, Chung-Ang University, Seoul 06974, Korea; yjkim@cau.ac.kr (Y.-J.K.); cheer1007@naver.com (J.-H.C.)

**Keywords:** COVID-19, Daegu, sense of belonging, pride, mental health, disaster relief fund

## Abstract

Korea’s Daegu Metropolitan City once had the second highest rate of COVID-19 infection after Wuhan in China. Following the outbreak, the government provided the first national disaster relief fund to citizens as financial aid. This study investigated whether the sense of regional belonging, pride, and mental health among 550 citizens of Daegu differed between the times before and after COVID-19, based on the presence or absence of the disaster relief fund. Frequency analysis, descriptive statistical analysis, and *t*-tests were conducted using the SPSS 25.0 program. Results showed that the sense of belonging was higher after COVID-19 than before, while pride was lower. Individuals who received the disaster relief fund showed higher levels of regional belonging and pride with statistical significance. The prevalence of melancholy and depression increased after COVID-19, but the presence or absence of the fund did not lead to a significant difference. Thus, in case of a future national disaster level, provision of the disaster relief fund can raise the sense of regional belonging and pride, in order to elicit communication among local residents toward overcoming difficulties. Furthermore, during challenging disaster situations, central and local governments should provide diverse programs for the citizens’ mental health care.

## 1. Introduction

The high infectivity of COVID-19, first confirmed in China at the end of 2019, led to an exponential increase in the rates of incidence and infection [1]. COVID-19 has now spread across the world, adversely affecting the political, economic, psychological, and social states in each country [2].

The government of the Republic of Korea recently raised the COVID-19 national crisis level to “serious” [3], and the World Health Organization (WHO) has declared COVID-19 a global pandemic [4].

For a certain period in Korea, the COVID-19 incidence rapidly fell in comparison to other countries, and the daily lives of people seemed to return to normal [5]. However, the 31st patient confirmed to have COVID-19 on 18 February 2020, in Daegu Metropolitan City (Daegu) in Korea led to approximately 5300 additional confirmed patients within 15 days [6]. This spread of infection resulted in the United States raising the travel alert level to the highest phase, at 4, to prohibit trips to Daegu [7]. The rapid spread of COVID-19, however, turned out not just to be a situation in Daegu, Korea; the large number of confirmed cases in New York in the United States and the sudden rise in mortality paralyzed the city [8], and led to shortage of mortuaries in the city’s hospitals [9].

Daegu in Korea was designated as a disaster area, due to the large-scale spread of COVID-19. For the first time, the city provided “disaster relief funds” to citizens after the outbreak of COVID-19 [10]. The funds were determined to prioritize the class that had difficulty receiving national welfare for COVID-19 [11].

However, the households that exceed the standard of the national health insurance premium by 100%, teaching staff and employees of public institutions are not eligible for disaster relief funds [12]. Daegu has offered prepaid cards, local currency, and gift cards that could only be used to help the financially distressed citizens and the local economy during the COVID-19 pandemic. This has been contributing to the revitalization of the local economy [13].

According to Park [14], overcoming regional economic difficulties is one of the ways of making citizens feel a sense of stability and a sense of belonging. Park [15] also stated that feeling proud of contributing to the restoration of a local market by using local currency leads to a sense of belonging to and affection for the community, and provides a chance to change the social atmosphere depressed by COVID-19 in a positive way.

The local residents of a city with a high number of confirmed COVID-19 cases may suffer from subsequent psychological trauma [16]. Such emotional damage could reduce residents’ sense of regional belonging, as well as solidarity [17]. Thus, the basic data of the residents’ sense of belonging and pride are necessary for those in regions, including Daegu, where COVID-19 incidence is high.

A terrifying aspect of COVID-19 is the ambiguity of the information regarding the factors related to the propagation, latency, geographical range, infection frequency, and actual mortality. In fact, an incorrect piece of information could induce anxiety and fear in people, which has a direct negative influence on mental health [18,19]. To understand the psychological and emotional impact of a pandemic, feelings like fear and rage have to be considered. The feeling of fear is a basic human psychological response to a potentially threatening incidence [20]. Nonetheless, continuing fear could exert harmful influence on mental health [21]. National disasters, such as the Ebola virus, severe acute respiratory syndrome outbreaks, and earthquakes, have been shown to have a negative influence on mental health [22,23,24]. When combating infectious diseases, fear increases the levels of anxiety and stress in healthy individuals, while aggravating the symptoms in those already suffering from a mental disorder, thus deteriorating their health further [25].

Quality of life and health are influenced by the balance between work and life, suggesting that quality of life could be enhanced when a healthy mind and lifestyle achieve harmony [26]. Hence, maintaining the quality of life at an adequate level is likely to improve the defense mechanism against stress and produce emotional stability [27].

A study on mental health among citizens of Hong Kong after the COVID-19 outbreak reported that mental health was influenced by COVID-19 [28]. The continuous spread of COVID-19 has resulted in a deterioration of the quality of life of Daegu citizens, and several people are experiencing reduced mental health [29]. Therefore, this study aimed to determine the necessary factors to aid people when a national disaster occurs, such as in the case of Daegu, while examining the needs of people during the spread of COVID-19. Furthermore, the sense of belonging, pride, and mental health as experienced by the citizens of Daegu was investigated, as Daegu is the city with the largest number of COVID-19 cases in Korea, and the differences based on the presence or absence of the national disaster relief fund was examined.

## 2. Materials and Methods

### 2.1. Study Design

This study analyzes the difference between the present and the past with group infection, based on the data of “Social Indices of Daegu in 2018” [30] and “Regional Health Statistics at a glance, 2008–2018” [31], published in the Daegu area before the COVID-19. A comparison between the presence and absence of national disaster relief fund. The data collection period was from 8 May to 13 May 2020.

### 2.2. Participants

A survey was conducted among Daegu residents aged 19–65 years, excluding adolescents and older adults. Convenience sampling was used, and a self-reported questionnaire was administered. The survey (550 sheets) was conducted online through EMBRAIN, the top survey company in Korea. Consent for participation was obtained from each participant using a preliminary question set, before conducting the survey. The study design was reviewed and approved by the Institutional Review Board at Chung-Ang University (1041078-202005-HRSB-119-01).

### 2.3. Measurement

To investigate the differences in pride, sense of belonging, and mental health among Daegu citizens with respect to COVID-19, the following measurement tools were used. The questionnaire required 10 min on average for completion.

#### 2.3.1. Indicators of Sense of Belonging and Pride

To measure regional pride of Daegu citizens and its levels, a scale was developed, based on “Social Indices of Daegu in 2018” [30]. The sub-categories of a sense of belonging and pride were comprised of a total of two questions: one question about a sense of belonging as a Daegu citizen and one question about satisfaction. Based on the four-point Likert scale, the scale of a sense of belonging and pride contains four sentences regarding a sense of belonging: “Very strong sense of belonging”, “A little sense of belonging”, “Little no sense of belonging”, and “No sense of belonging at all”. There were also four sentences regarding pride: “Very strong sense of pride”, “A little sense of pride”, “Little no sense of pride”, and “No sense of pride at all”.

#### 2.3.2. Indicators of Mental Health

Indicators of the mental health status of the citizens were based on the “Regional Health Statistics at a glance, 2008–2018” [31] survey conducted by the Ministry of Health and Welfare and the Korea Centers for Disease Control. The sub-category of mental health was comprised of one question on average daily sleep time and stress awareness, one question on the experience of the feeling of melancholy, and nine questions on the prevalence of depression. The sleep time diagnosis consisted of average daily sleep time was measured. The stress was composed of a four-point scale, and the degree of stress perceived by individuals was indicated. The melancholy experience was usually composed of yes or no, as a question about melancholy. Among the questions, the one regarding depression prevalence used the standardized Korean version of the nine-item Patient Health Questionnaire (PHQ-9), which is a screening tool for depression comprised of nine questions, based on the Diagnostic and Statistical Manual of Mental Disorders, 4th edition [32]. Based on the diagnosis of the prevalence of depression, 0 = “not at all”, 1 = “several days”, 2 = “more than half the days”, and 3 = “nearly every day” were the criteria used to diagnose the prevalence of depression. The total score ranged 0 from 27, and a higher score means a higher incidence of depression [32]. The tool has been reported to have better sensitivity (88%) and specificity (88%) than the Self-Rating Depression Scale and Beck Depression Inventory, which are the most commonly used depression indicators. PHQ-9 has also been found to be valid, with a high level of reliability in the measurement of the severity of depression [33]. Despite the small number of questions that require less time for completion, the PHQ-9 has been reported as a more accurate diagnostic tool for depression in numerous recent studies [34]. This assumes significance in the present time, when almost half of the patients with depression remain undiagnosed, owing to insufficient consultation time in overcrowded outpatient clinics, or wherein most depression patients are examined primarily for physical symptoms like pain or reduced physical functions, rather than psychological ones [35]. In the present study, the Cronbach’s alpha coefficient of the PHQ-9 was 0.88.

### 2.4. Data Analysis

All data in this study were analyzed using SPSS version 25.0 (IBM, Armonk, NY, USA), after a process of coding and data cleaning. For demographic factors, cross-analysis and descriptive statistical analysis were performed, and the results compared in order to analyze the mean and standard deviation from the “Social Indices of Daegu in 2018” and the “Regional Health Statistics at a Glance, 2008–2018”. to ensure the required level of confidence in each tool, Cronbach’s α values were computed. Lastly, in order to identify the differences according to provision of the national disaster relief fund, an independent *t*-test was carried out.

## 3. Results

Table 1 displays the characteristics of the participants. This study targeted 285 females (51.8%) and 265 males (48.2%), which means more females participated in the study. Also, 138 people in their 20s (25.1%) occupied the highest proportion of the subjects, and it was followed by 137 in their 40s (24.9%). There were statistically significant differences in age, monthly household income, and sense of pride depending on national disaster relief funds. Also, there were significant differences in national disaster relief funds depending on the PHQ-9 scores of the subjects.

Table 2 includes a descriptive statistics analysis to determine the sleep time, stress level, melancholy and depression prevalence, sense of belonging, and sense of pride among Daegu citizens before and during COVID-19. Looking at the results, it was found that in 2016, the sleep time was at 6.6 h in 2016 and 6.8 h in the case of COVID-19. The stress level was 23.3% in 2018 and 42.4% with the current COVID-19. The depression was at 3.4% in 2018 and at 18.9% during COVID-19. The prevalence of depression was 1.9% in 2018 and 5.8% as of the emergence of COVID-19.

For the sense of belonging as a citizen of Daegu, “Very strong sense of belonging” was the lowest, at 5.9% in 2018—as of COVID-19, it was 12.4%. “A little sense of belonging” was at 47.2% in 2018, and 51.3% as of COVID-19. “Little no sense of belonging” was at 36.9% in 2012 and 2016, and 29.6% during COVID-19. “No sense of belonging at all” was at 2.0% in 2012, and 6.7% with COVID-19. Regarding pride, the “Very strong sense of pride” response was the lowest, at 5.6% in 2016 and currently at 7.5% during COVID-19. “A little sense of pride” was at 39.8% in 2018, and 29.6% during COVID-19. “Little no sense of pride” was at 43.0% in 2016, and COVID-19 responses were at 47.1%. Lastly, “No sense of pride at all” had 3.2% in 2012 and 15.8% with COVID-19.

Table 3 shows the independent *t*-test to determine whether each parameter showed differences according to provision of the disaster relief fund. It shows that all parameters exhibited differences between the presence and absence of the fund (sense of pride: *t* = 3.06, *p* = *0*.002; sense of belonging: *t* = 2.01, *p* = *0*.045).

The independent *t*-test to determine whether each parameter showed differences, according to provision of the disaster relief fund, showed that none of the parameters showed a difference between presence and absence of the fund, with the following results: depression prevalence, *t =* 1.01, *p* = 0.312; stress levels, *t* = –0.454, *p* = 0.650; and melancholy, *t* = –0.106, *p* = 0.269.

## 4. Discussion

This study aimed to investigate whether COVID-19 had led to differences in the sense of regional belonging, pride, and mental health among the citizens of Daegu, and to analyze the differences based on the presence or absence of the national disaster relief fund. The findings are discussed as follows.

First, the sense of regional belonging was found to have increased after COVID-19 when compared to the time before. Daegu was pointed out as the source of the explosive spread of COVID-19 by the general public, who criticized the citizens of Daegu and posted various expressions of hate on online portal sites and communities, holding them responsible for the COVID-19 outbreak incessantly [36]. Such accusations and discriminating behaviors from people in other regions are thought to have led to the increased sense of belonging among the people of Daegu. In the same context, as the people in other regions blamed the citizens of Daegu, the increased social sharing among the local residents enhanced the regional identity, and subsequently, the regional belonging. Daegu has endured a number of disasters in the past, such as a gas explosion and metro fire, and in each case, the disaster was overcome by the united efforts of the citizens [37]. This is presumed to be the reason behind the increased sense of regional belonging among the citizens of Daegu after COVID-19.

Individuals who received the national disaster relief fund were found to have higher awareness of regional belonging. Financial difficulties in households are being relieved by the disaster relief fund provided by the government [38]. It is thus presumed that the fund is financially aiding small business owners and socially disadvantaged groups to help them overcome the difficult time [39], and individuals who received the fund are consequently showing higher awareness of regional belonging [40]. The disaster relief fund was reported to have a positive influence on the quality of life of the working poor and people with disabilities, who are able to make less than half their original profits owing to the COVID-19 outbreak [41]. Thus, in a prolonged national disaster situation, as is the case of COVID-19, with consequently reduced social and economic activities, providing the national disaster relief fund to citizens, starting with small business owners and the socially disadvantaged, was found to have led to higher awareness of regional belonging among the citizens. Such an increase in regional belonging is likely to strengthen the solidarity among local residents, and the resulting social unity would help the citizens overcome the difficult situation, based on the strong confidence regarding the region, in case of a national disaster in the future.

The regional pride among the citizens of Daegu was shown to have decreased from 2018 after the arrival of COVID-19. This finding contradicts a previous study [42]. It has been reported that the number of additional confirmed patients have substantially decreased in Daegu, Korea—a city that had faced the first phases of COVID-19 outbreak outside China. In reality, however, Daegu is viewed by its citizens as a city where such a social difficulty or disaster may occur again at any time, even though might have overcome the COVID-19 situation, which consequently led to the fall in their regional pride.

This study shows that individuals who received national disaster relief funds had a higher level of pride. According to a previous study, the people of Daegu show high levels of unity, justice, and resourcefulness [43]. This is thought to have increased an already high level of regional pride among the citizens when the national disaster relief fund was provided. Thus, based on an already high level of regional pride, the sense of community among the citizens of Daegu is likely to have increased, and the fund is thought to have further increased their regional pride. Thus, the national disaster relief fund provided to the citizens of Daegu has led to trust towards the region, owing to regional characteristics like conservative ideas, loyalty, and exclusivity; such trust may serve as the basis for communication among local residents, which would help characterize Daegu as a city that can overcome any disaster in the future.

The rate of experiencing melancholy and depression among the citizens of Daegu after COVID-19 was shown to have increased. In addition, no significant difference was found for these parameters with respect to provisions of the disaster relief fund, based on the results of the *t*-test. This indicates that the presence or absence of the fund did not have a significant impact on people’s mental health, and that COVID-19 itself is presumed to have had a negative influence on the mental health of the citizens. Hence, the reduced mental health compared to the past is thought to have resulted from the social isolation, staying indoors, and altered daily activities caused by COVID-19. Indicators such as melancholy, depression, and anxiety among the citizens of Hong Kong after COVID-19 showed far higher values than the time before, which is consistent with the results in this study [28]. The continuous increase in the level of melancholy due to COVID-19 among Daegu citizens has even led to coining of the term “Corona Blues”, combining the coronavirus and melancholy (blues) [44]. The level of melancholy is thought to have increased in line with the increased anxiety among Daegu citizens, due to the rapid spread of COVID-19, which once led to as high as 741 confirmed patients a day. The social distancing from friends and family due to COVID-19 has led many people to suffer from melancholy [45]. In addition, the changed economic and social values and relationships after COVID-19 are exerting an influence on the psychological stress in people [46]. The findings of these previous studies were partially in agreement with those of the present study. Uncertain situations about COVID-19 have resulted in increased stress and anxiety about not maintaining daily life [47]. In the future, therefore, when a focused disaster situation occurs in a single city as in the case of Daegu, various methods should be sought to reduce the levels of melancholy and stress among the citizens for their mental health care through the use of different media, including phone and the internet, or through a national program developed for the emotional stability of the citizens.

This study has several limitations, as follows. First, the data of the study were gathered online with the use of a professional survey company. For this reason, the way of responding might be influenced by using a computer and computer skills. Second, this is a cross-sectional study, which means it is impossible to observe temporary changes in respondents’ sense of belonging, pride, and mental health. Third, the study results cannot apply to other regions and groups in Korea for generalization. Fourth, although giving national disaster relief funds may influence a sense of belonging and pride, these can be boosted by more diverse psychological and social factors. That is why it is impossible to generalize the levels of a sense of belonging and pride revealed in this study.

## 5. Conclusions

This study examined the differences in the sense of regional belonging, pride, and mental health among the citizens of Daegu, due to COVID-19, between the times before and after COVID-19, and based on the presence or absence of the national disaster relief fund.

The level of regional belonging among the citizens increased after COVID-19, but the level of pride decreased. Regarding differences according to provision of the national disaster relief fund, all parameters of belonging and pride significantly increased among individuals who received the fund. This was thought to be due to the enhanced regional identity, based on the characteristics of Daegu citizens, which involve conservative ideas, local patriotism, loyalty, exclusivity, and faithfulness, combined with the social convention to overcome a difficult situation together. Therefore, in a challenging national situation, providing the disaster relief fund to the citizens is anticipated to raise the sense of regional belonging and pride to enhance regional identity and elicit communication among local residents, whereby the challenge can be overcome.

Furthermore, the citizens of Daegu were found to have experienced melancholy more frequently after COVID-19, and the depression prevalence was also higher compared to the time before COVID-19. In line with this, to protect the mental health of individuals in a national disaster, such as COVID-19, every local government should take initiatives to enhance the mental health of the citizens, and in a narrower sense, a way to enhance the mental health should be sought through the provision of aids to each household, so that the family members can acquire emotional stability.

## Figures and Tables

**Table 1:** Demographic characteristics (*n* = 550).

Variable	Total	WDRF ^1^	WODRF ^2^	*p*-Value
*n*	550	264	286	
Gender				
Male, *n* (%)	265 (48.2)	128 (48.3)	137 (51.7)	0.891
Female, *n* (%)	285 (51.8)	136 (47.7)	149 (52.3)	
Age, mean (SD)	38.33 (10.64)	38.09 (10.66)	38.54 (10.64)	
20s, *n* (%)	138 (25.1)	69 (50.0)	69 (50.0)	
30s, *n* (%)	190 (34.5)	88 (46.3)	102 (53.7)	
40s, *n* (%)	137 (24.9)	73 (53.3)	64 (46.7)	0.017
50s, *n* (%)	66 (12.0)	21 (31.8)	45 (68.2)	
60–65 years *n* (%)	19 (3.5)	13 (68.4)	6 (31.6)	
Marital Status				
Single, *n* (%)	285 (51.8)	131 (46.0)	154 (54.0)	
Married, *n* (%)	249 (45.3)	123 (49.4)	126 (50.6)	0.365
Widowed or divorced, *n* (%)	16 (2.9)	10 (62.5)	6 (37.5)	
Education				
Under high school, *n* (%)	52 (9.5)	29 (55.8)	23 (44.2)	
College (2-year), *n* (%)	124 (22.5)	62 (50.0)	62 (50.0)	
University (4-year), *n* (%)	307 (55.8)	149 (48.5)	158 (51.5)	0.228
Master’s degree, *n* (%)	54 (9.8)	20 (37.0)	34 (63.0)	
PhD degree, *n* (%)	13 (2.4)	4 (30.8)	9 (69.2)	
Monthly household income ^a^				
Under ₩ 1,000,000, *n* (%)	22 (4.0)	17 (77.3)	5 (22.7)	
₩ 1,000,000–1,990,000, *n* (%)	32 (5.8)	23 (71.9)	9 (28.1)	
₩ 2,000,000–2,990,000, *n* (%)	118 (21.5)	68 (57.6)	50 (42.4)	
₩ 3,000,000–3,990,000, *n* (%)	90 (16.4)	47 (52.2)	43 (47.8)	0.000 *
₩ 4,000,000–4,990,000, *n* (%)	84 (15.3)	35 (41.7)	49 (58.3)	
₩ 5,000,000–5,990,000, *n* (%)	86 (15.6)	39 (45.3)	47 (54.7)	
₩ 6,000,000–6,990,000, *n* (%)	50 (9.1)	17 (34.0)	33 (66.0)	
₩ ≥ 7,000,000, *n* (%)	68 (12.4)	18 (16.5)	50 (73.5)	
Sleep time, mean (SD)	6.77(1.16)	6.77 (1.20)	6.76 (1.13)	0.946
Stress levels				
I feel a lot of stress frequently	57 (10.4	28 (49.1)	29 (50.9)	
I feel stress a lot	176 (32.0)	84 (47.7)	92 (52.3)	0.713
I feel stress a little	281 (51.1)	138 (49.1)	143 (50.9)	
I do not feel stress	36 (6.5)	14 (38.9)	22 (61.1)	
Melancholy				
Yes	104 (18.9)	55 (52.9)	49 (47.1)	0.268
No	446 (81.1)	209 (46.9)	237 (53.1)	
PHQ-9				
PHQ-9 score 0-4 (minimal)	266 (48.4)	117 (44.0)	149 (56.0)	
PHQ-9 score 5–9 (mild)	173 (31.5)	88 (50.9)	85 (49.1)	
PHQ-9 score 10–14 (moderate)	77 (14.0)	46 (59.7)	31 (40.3)	0.004
PHQ-9 score 15–19 (moderately severe)	23 (4.2)	5 (21.7)	18 (78.3)	
PHQ-9 score 20–27 (severe)	11 (2.0)	8 (72.7)	3 (27.3)	
Sense of belonging				
Very strong sense of belonging	68 (12.4)	36 (52.9)	32 (47.1)	
A little sense of belonging	282 (51.3)	141 (50.0)	141 (50.0)	0.174
Little no sense of belonging	163 (29.6)	75 (46.0)	88 (54.0)	
No sense of belonging at all	37 (6.7)	12 (32.4)	25 (67.6)	
Sense of pride				
Very strong sense of pride	41 (7.5)	27 (65.9)	14 (34.1)	
A little sense of pride	163 (29.6)	82 (50.3)	81 (49.7)	0.012
Little no sense of pride	259 (47.1)	124 (47.9)	135 (52.1)	
No sense of pride at all	87 (15.8)	31 (35.6)	56 (64.4)	

Notes: ^1^ WDRF: with disaster relief fund; ^2^ WODRF: without disaster relief fund; ^a^ South Korean 10.000 won (USD 1 = KRW 1203.00). The *p*-values were calculated from chi-square tests and *t*-tests, as appropriate. * Significant difference noted in the comparison between four groups (*p* < 0.05).

**Table 2:** The mean and standard deviation (SD) of the mental health indicators, sense of belonging, and pride among Daegu citizens before and during COVID-19 (*n* = 550).

Items	2012	2014	2016	2018	COVID-19
Sleep time (hours), mean (SD)	6.7	6.7	6.6	6.7	6.8 (1.16)
Stress levels, % (*n*)	27.2	26.0	26.6	23.3	42.4 (233)
Melancholy, mean (SD)	5.6	5.4	4.9	3.4	18.9 (0.39)
Depression prevalence, mean (SD)				1.9	5.8 (5.05)
Sense of belonging					
Very strong sense of belonging, % (*n*)	9.9	8.3	7.1	5.9	12.4 (68)
A little sense of belonging, % (*n*)	51.2	49.2	50.8	47.2	51.3 (282)
Little no sense of belonging, % (*n*)	36.9	38.7	36.9	41.2	29.6 (163)
No sense of belonging at all, % (*n*)	2.0	3.8	5.2	5.8	6.7 (37)
Sense of pride					
Very strong sense of pride, % (*n*)	7.7	7.5	5.6	5.9	7.5 (41)
A little sense of pride, % (*n*)	47.2	43.0	45.8	39.8	29.6 (163)
Little no sense of pride, % (*n*)	42.0	45.5	43.0	48.0	47.1 (259)
No sense of pride at all, % (*n*)	3.2	4.1	5.6	6.3	15.8 (87)

**Table 3:** The differences between presence and absence of the national disaster relief fund (*n* = 550).

Items	Mean (SD)		Effect Size	*t*-Value	*p*-Value
	WDRF ^1^ (*n* = 264)	WODRF ^2^ (*n* = 286)			
PHQ-9, mean (SD)	1.85 (0.97)	1.74 (0.95)	0.12	1.40	0.163
Depression prevalence *	1.67 (0.57)	1.62 (0.55)	−0.09	1.01	0.312
Stress levels *	2.52 (0.76)	2.55 (0.78)	−0.04	−0.45	0.650
Melancholy *	1.79 (0.41)	1.83 (0.38)	−0.10	−0.10	0.269
Sense of belonging	2.76 (0.74)	2.62 (0.80)	0.17	2.01	0.045
Sense of pride	2.39 (0.83)	2.18 (0.80)	0.26	3.06	0.002

Notes: *p*-values were analyzed using the independent *t*-test. ^1^ WDRF: with disaster relief fund; ^2^ WODRF: without disaster relief fund. * Depression prevalence, stress levels, and melancholy each showed no significant difference.
